# Characterization of Effectiveness in Concerted *I*_h_ Inhibition and *I*_K(Ca)_ Stimulation by Pterostilbene (Trans-3,5-dimethoxy-4′-hydroxystilbene), a Stilbenoid

**DOI:** 10.3390/ijms21010357

**Published:** 2020-01-05

**Authors:** Edmund Cheung So, Zi-Han Gao, Shun Yao Ko, Sheng-Nan Wu

**Affiliations:** 1 Department of Anesthesia and Medical Research, An Nan Hospital, China Medical University, Tainan 70965, Taiwan; edmundsotw@gmail.com; 2 Graduate Institute of Medical Sciences, Chang Jung Christian University, Tainan 71101, Taiwan; 3 Department of Physiology, National Cheng Kung University Medical College, No. 1, University Road, Tainan 70101, Taiwan; hhelen000111tw@gmail.com; 4 Institute of Basic Medical Sciences, National Cheng Kung University Medical College, Tainan 70101, Taiwan; 5 Department of Medical Research, China Medical University Hospital, China Medical University, Taichung 40402, Taiwan

**Keywords:** Pterostilbene (trans-3,5-dimethoxy-4′-hydroxystilbene), hyperpolarization-activated cation current, Ca^2+^-activated K^+^ current, large-conductance Ca^2+^-activated K^+^ channel, pituitary cell, hippocampal neuron

## Abstract

Pterostilbene (PTER), a natural dimethylated analog of resveratrol, has been demonstrated to produce anti-neoplastic or neuroprotective actions. However, how and whether this compound can entail any perturbations on ionic currents in electrically excitable cells remains unknown. In whole-cell current recordings, addition of PTER decreased the amplitude of macroscopic *I*_h_ during long-lasting hyperpolarization in GH_3_ cells in a concentration-dependent manner, with an effective IC_50_ value of 0.84 μM. Its presence also shifted the activation curve of *I*_h_ along the voltage axis to a more hyperpolarized potential, by 11 mV. PTER at a concentration greater than 10 μM could also suppress l-type Ca^2+^ and transient outward K^+^ currents in GH_3_ cells. With the addition of PTER, *I*_K(Ca)_ amplitude was increased, with an EC_50_ value of 2.23 μM. This increase in *I*_K(Ca)_ amplitude was attenuated by further addition of verruculogen, but not by tolbutamide or TRAM-39. Neither atropine nor nicotine, in the continued presence of PTER, modified the PTER-stimulated *I*_K(Ca)_. PTER (10 μM) slightly suppressed the amplitude of l-type Ca^2+^ current and transient outward K^+^ current. The presence of PTER (3 μM) was also effective at increasing the open-state probability of large-conductance Ca^2+^-activated K^+^ (BK_Ca_) channels identified in hippocampal mHippoE-14 neurons; however, its inability to alter single-channel conductance was detected. Our study highlights evidence to show that PTER has the propensity to perturb ionic currents (e.g., *I*_h_ and *I*_K(Ca)_), thereby influencing the functional activities of neurons, and neuroendocrine or endocrine cells.

## 1. Introduction

Pterostilbene (PTER, trans-3,5-dimethoxy-4′-hydroxystilbene) is a natural dimethylated analog of resveratrol and was named after a natural phenolic compound found in *Pterocarpus marsupium* Roxb. (Fabaceae), which is native to India, Nepal, and Sri Lanka, and is also one of the active compounds in the extracts of *P. marsupium* that were used in Ayurvedic medicine for the treatment of various disorders [1]. This compound has been reported to have benefits for the prevention or treatment of different kinds of cancers, as mounting evidence has demonstrated its inhibitory effects on almost every cellular event that promotes tumor progression toward metastasis in apoptosis-dependent or apoptosis-independent manners [2,3,4,5,6,7,8,9,10,11,12,13].

Recent evidence has revealed that PTER could exert protective actions against the oxidative stress or inflammatory reaction in various types of neurons, such as neuronal SH-SY5Y cells and spinal cord neurons, as well as in ischemia- or hypoxia-induced brain injuries [14,15,16,17,18,19,20,21]. PTER and other resveratrol analogs were previously reported to exert antiepileptic actions [22,23,24] to decrease dopamine release in striatal slices, and to substantially ameliorate depressive or cognitive behavioral deficits in rats [25,26,27]. It is also noted that the presence of PTER could disrupt pituitary function [3,28]. However, whether this agent is capable of perturbing different types of membrane ionic currents in central neurons is not thoroughly investigated, although resveratrol, a phytoalexin, has previously been demonstrated to modify large-conductance Ca^2+^-activated K^+^ (BK_Ca_) channels and voltage-gated Na^+^ currents in various types of cells including vascular endothelial cells, cardiac fibroblasts and cortical neurons [29,30,31].

Hyperpolarization-activated cation current (*I*_h_) has increasingly been considered to be an important determinant of repetitive electrical activity inherent in heart cells and in a variety of neurons, and neuroendocrine or endocrine cells [32,33,34,35,36,37,38,39]. This current is a mixed inward Na^+^/K^+^ current, which is sensitive to blocking by different compounds such as CsCl, ivabradine or zatebradine [40,41], and increased amplitude of its own accord can act to depolarize membrane potential to the threshold required for elicitation of action potential (AP) [33,35,36,37,42]. The current is regarded to be carried by channels of the hyperpolarization-activated cyclic nucleotide-gated (HCN) gene family, which belongs to the superfamily of voltage-gated K^+^ channels and cyclic nucleotide-gated channels [36,43]. Notably, the magnitude of *I*_h_ existing in central neurons has been burgeoningly reported to be intimately linked either to spatial working memory in behaving mice or in patients with schizophrenia, or to cognitive dysfunction [25,44,45,46,47,48]. However, how PTER or other stilbenoids can directly and functionally interact with the activity of HCN channels to modify the amplitude and gating of *I*_h_ remains largely unanswered.

In the current study, we intended to explore the perturbating effects of PTER on different types of ionic currents (e.g., hyperpolarization-activated cation current [*I*_h_], l-type Ca^2+^ current [*I*_Ca,l_], transient outward K^+^ current [*I*_K(TO)_], and Ca^2+^-activated K^+^ current [*I*_K(Ca)_]) in pituitary GH_3_ cells. Its effect on large-conductance Ca^2+^-activated K^+^ [BK_Ca_] channels in hippocampal mHippoE-14 neurons [4] was also examined. Findings from the present results highlight evidence to reveal that the addition of PTER is capable of suppressing *I*_h_ but stimulating *I*_K(Ca)_ in a concentration-dependent manner. Those actions on the activity of ionic currents could concertedly and substantially influence the functional activities of different electrically excitable cells (e.g., GH_3_ cells and mHippoE-14 neurons).

## 2. Results

### 2.1. Depressant Effect of PTER on Hyperpolarization-Activated Cation Current (I_h_) Identified in Pituitary GH_3_ Cells

**Figure 1:**
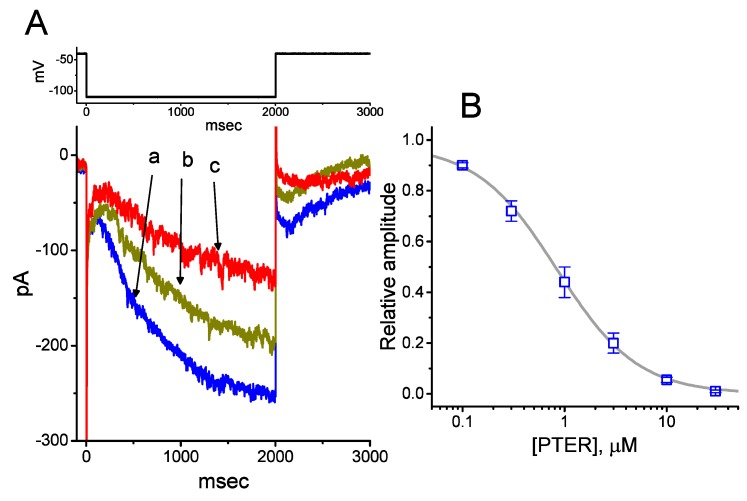
Effect of pterostilbene (PTER) on hyperpolarization-activated cation current (*I*_h_) taken from pituitary GH_3_ cells. In these experiments, we immersed cells in Ca^2+^-free Tyrode’s solution containing 1 μM tetrodotoxin (TTX), and the recording pipette used was filled with a K^+^-containing solution. The composition of these solutions is described under Materials and Methods. (**A**) Representative records of macroscopic *I*_h_ achieved under control conditions (i.e., PTER was not present, a) and during cell exposure to 0.3 μM PTER (b) and 1 μM PTER (c). The upper part is the voltage profile delivered. (**B**) Concentration–response relationship for PTER-induced inhibition of *I*_h_ amplitude activated during step hyperpolarization (mean ± SEM; *n* = 8 for each data point). Current amplitude was obtained at the end of 2-s hyperpolarizing step −110 mV from a holding potential of −40 mV. The bold sigmoidal curve indicates the best fit to the Hill equation, as detailed under Materials and Methods. The estimated values for IC_50_ and the Hill coefficient were 0.84 μM and 1.1, respectively.

We initially evaluated whether the *I*_h_ observed in these cells [34,38,39] was subject to modification by PTER. Whole-cell experiments were conducted in cells that were suspended in Ca^2+^-free Tyrode’s solution, and the recording pipette used was filled with a K^+^-containing solution. Within 1 min of exposing cells to different concentrations of PTER, the amplitude of *I*_h_ activated during 2-s membrane hyperpolarization to −110 mV from a holding potential of −40 mV was progressively reduced (Figure 1A). For example, the addition of PTER (1 μM) substantially decreased *I*_h_ amplitude from 255 ± 18 to 118 ± 12 pA (*n* = 9, *p* < 0.05). After washout of the agent, current amplitude returned to 249 ± 17 pA (*n* = 8, *p* < 0.05). At the same time, the addition of PTER at a concentration of 0.3 or 1 μM noticeably increased the activation time constant (τ) of *I*_h_ during 2-s step hyperpolarization to 811 ± 19 ms (*n* = 8, *p* < 0.05) or 931 ± 23 ms (*n* = 8, *p* < 0.05), respectively, from a control value of 628 ± 15 ms (*n* = 8).

**Figure 2:**
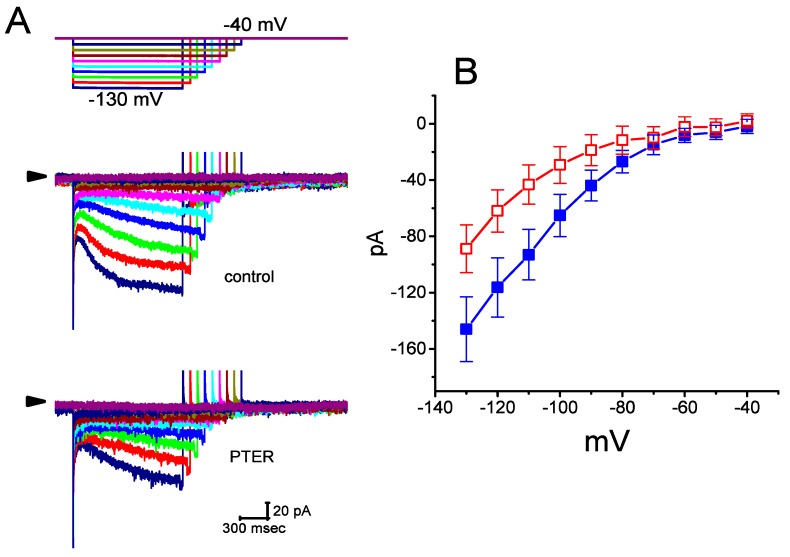
Effect of PTER on the current–voltage (*I*–*V*) relationships of *I*_h_ in GH_3_ cells. Current traces were recorded during 2-s voltage steps to a family of membrane potentials ranging between −130 and −40 mV in 10-mV steps from a holding potential of −40 mV, as indicated in the uppermost part of (**A**). (**A**) Representative *I*_h_ traces achieved under the control conditions (i.e., PTER was not present) (upper) and during the exposure to 1 μM PTER (lower). The arrowhead in each panel depicts the zero-current level, and the calibration mark shown in the right lower side applies to all current traces. Of note, there are varying durations in the voltage-clamp profile for better illustration. (**B**) Averaged *I*–*V* relations of *I*_h_ achieved in the absence (■) and presence (□) of 1 μM PTER (mean ± SEM; *n* = 9 for each data point). Current amplitude was measured at the end of each voltage step.

Figure 1B illustrates that PTER can depress the amplitude of *I*_h_ activated during step hyperpolarization in a concentration-dependent fashion. The IC_50_ value for PTER-mediated suppression of *I*_h_ identified in GH_3_ cells was 0.84 μM. However, in the continued presence of PTER 1 μM, subsequent addition of atropine (10 μM) or nicotine (10 μM) to the bath medium was unable to attenuate the PTER-mediated inhibition of *I*_h_. For example, the *I*_h_ amplitude did not differ between PTER (1 μM) alone and PTER (1 μM) plus atropine (10 μM) (133 ± 15 pA [PTER alone] versus 132 ± 16 pA [PTER plus atropine], *n* = 7, *p* > 0.05). The current–voltage (*I*–*V*) relationships of *I*_h_ established at various levels of hyperpolarizing steps were also constructed and are depicted in Figure 2. It was noted that the presence of PTER (1 μM) substantially reduced the slope of the linear fit of *I*_h_ amplitudes to the voltages between −130 and −100 mV from 27.2 ± 1.3 to 13.1 ± 1.1 nS (*n* = 8, *p* < 0.05). The results thus demonstrate that PTER has a conceivable depressant action on *I*_h_ functionally expressed in GH_3_ cells.

### 2.2. Effect of PTER on the Activation Curve of I_h_ Recorded from GH_3_ Cells

**Figure 3:**
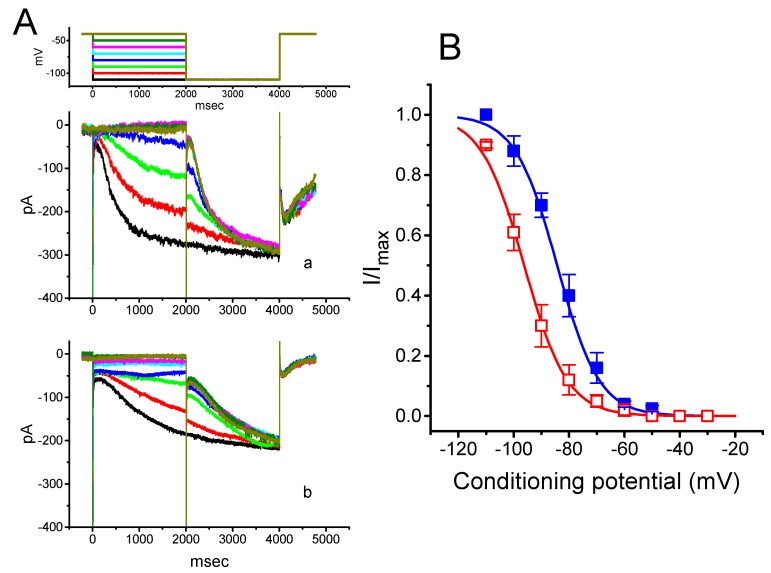
Effect of PTER on the quasi-steady state activation curve of *I*_h_ recorded from GH_3_ cells. The experiments were conducted with a two-step voltage pulse. The conditioning potentials had a duration of 2 s to the voltage ranging from −110 to −30 mV in 10-mV steps. Following each conditioning potential, a 2-s hyperpolarizing pulse to −110 mV was applied to activate *I*_h_. (**A**) Representative *I*_h_ traces evoked during this two-step voltage protocol (indicated in the uppermost part of (**A**)). Current records labeled “a” are controls, and those labeled “b” were taken during cell exposure to 1 μM PTER. (**B**) Steady-state activation curves of *I*_h_ obtained in the absence (■) and presence (□) of 1 μM PTER (mean ± SEM; *n* = 9) (9 for each data point). Notably, as the cells were exposed to PTER, there was a shift in the curve along the voltage axis toward more hyperpolarized potentials by 11 mV; however, no conceivable change in the gating charge of the curve was demonstrated.

To further characterize the inhibitory effect of PTER on *I*_h_, we investigated the voltage dependence of its effect on *I*_h_ in these cells. Figure 3 shows the quasi-steady state activation curve of *I*_h_ with or without addition of 1 μM PTER. These experiments were conducted with a two-step voltage pulse profile, that is, a 2-s conditioning pulse to various membrane potentials (i.e., the voltage between −110 and −30 mV in 10-mV steps) preceded the test pulse (2 s in duration) to −110 mV from a holding potential of −40 mV. To ensure complete recovery of *I*_h_, the duration between two sets of voltage pulses applied was set at 60 s.

The relationships between the normalized amplitudes of *I*_h_ and the conditioning potentials obtained with or without PTER (1 μM) addition were plotted and the data sets were thereafter approximately fitted to a Boltzmann function, as described in Materials and Methods. In the control, *V*_1/2_ = −83.5 ± 1.9 mV, and *q* = 3.07 ± 1.8 *e* (*n* = 9), whereas during exposure to 1 μM PTER, *V*_1/2_ = −94.2.5 ± 2.1 mV, and *q* = 3.07 ± 1.7 *e* (*n* = 9). Therefore, it is clear from these data that the presence of PTER did not merely suppress the maximal conductance of *I*_h_, but also was able to significantly shift the activation curve of the current to a hyperpolarized potential by approximately 11 mV. However, we failed to find any change in the gating charge of the curve achieved during cell exposure to PTER. The results indicate that, besides the reduction of *I*_h_ amplitude during long-lasting step hyperpolarization, PTER can alter the voltage dependence of *I*_h_ in these cells.

### 2.3. Effect of PTER on l-Type Ca^2+^ Current (I_Ca,l_) in GH_3_ Cells

**Figure 4:**
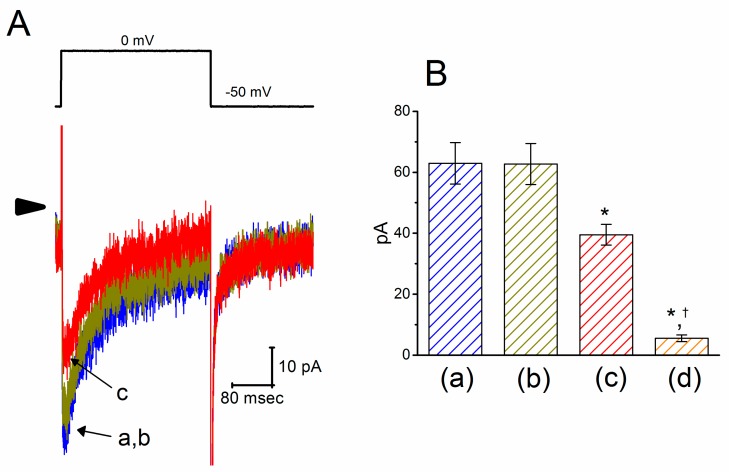
Inhibitory effect of PTER on l-type Ca^2+^ current (*I*_Ca,l_) identified in GH_3_ cells. This set of experiments was conducted in normal Tyrode’s solution, which contained 1.8 mM CaCl_2_, 10 mM tetraethylammonium chloride (TEA), and 1 μM TTX. (**A**) Original *I*_Ca,l_ traces evoked during 300-ms depolarizing pulse to 0 mV from a holding potential of −50 mV (indicated in the upper part). The arrowhead depicts the zero-current level, and the calibration mark in the right lower side applies to all current traces. The current trace labeled “a” is control, and that labeled “b” or “c” was obtained during exposure to 3 or 10 μM PTER, respectively. (**B**) Summary bar graph showing the effects of PTER and PTER plus SDZ-202791 on the peak amplitude of *I*_Ca,l_ in GH_3_ cells (mean ± SEM; *n* = 8–9 for each bar). Current amplitude was measured at the beginning of each depolarizing pulse from −50 to 0 mV. a: control; b: 3 μM PTER; c: 10 μM PTER; d: 10 μM PTER plus 3 μM SDZ-202791. *Significantly different from control (*p* < 0.05); †significantly different from the PTER (10 μM) alone group (*p* < 0.05).

We next evaluated any possible perturbations of this agent on *I*_Ca,l_ previously described in these cells [49]. In these experiments, cells were bathed in normal Tyrode’s solution containing 1.8 mM CaCl_2_, 1 μM tetrodotoxin (TTX) and 10 mM tetraethylammonium chloride (TEA), and the pipette used was filled with a Cs^+^-containing solution. As shown in Figure 4, the peak amplitude of *I*_Ca,l_ activated during step depolarization to 0 mV from a holding potential of −50 mV was decreased in the presence of PTER (10 μM) to 39.5 ± 3.4 pA (*n* = 9, *p* < 0.05) from a control value of 62.9 ± 6.8 pA (*n* = 9). Moreover, subsequent addition of SDZ-202791 (3 μM), an inhibitor of *I*_Ca,l_ [50], but still in the continued presence of 10 μM PTER, further decreased the peak *I*_Ca,l_ to 5.5 ± 1.1 pA (*n* = 8, *p* < 0.05).

### 2.4. Mild Inhibition by PTER of Transient Outward K^+^ Current (I_K(TO)_) Recorded from GH_3_ Cells

**Figure 5:**
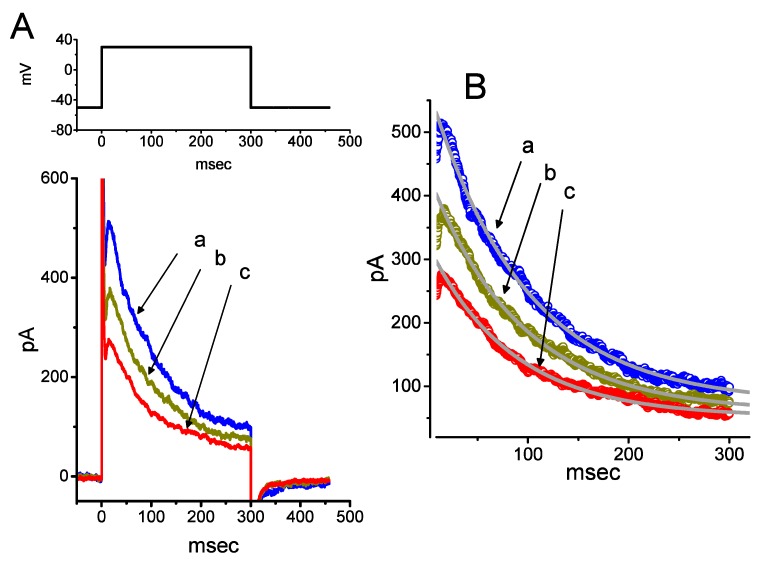
Inhibitory effect of PTER on transient outward K^+^ current (*I*_K(TO)_) in GH_3_ cells. Cells were bathed in Ca^2+^-free Tyrode’s solution, and the recording pipette was filled with K^+^-containing solution containing 1 μM TTX. (**A**) Superimposed *I*_K(TO)_ traces acquired in the control (a) and after the addition of 10 μM PTER (b) or 30 μM PTER (c). The upper part indicates the voltage protocol applied, i.e., the holding potential was −50 mV and clamp pulses to +50 mV with a duration of 300 ms were applied. (**B**) Effect of PTER on the inactivation time course of *I*_K(TO)_ elicited during 300-ms membrane depolarization. The bold approximate curves are nonlinear least squares fits of a single exponential to the records. a: control; b: 10 μM PTER; c: 30 μM PTER. The values of the inactivation time constant (τ) for *I*_K(TO)_ labeled “a”, “b” and “c” were estimated to be 94.8, 91.2 and 83.1 ms, respectively.

We continued to determine whether PTER could alter another type of K^+^ current, i.e., *I*_K(TO)_. This set of whole-cell current recordings was conducted in cells bathed in Ca^2+^-free Tyrode’s solution, and the pipette was filled with K^+^-containing solution. However, unlike its modulatory effects on *I*_h_ or *I*_K(Ca)_ mentioned above, *I*_K(TO)_ was relatively resistant to being altered by the presence of PTER (3 μM). However, PTER at a concentration higher than 10 μM significantly suppressed the amplitude of *I*_K(TO)_, accompanied by a slowing in the inactivation time course of the current (Figure 5). For example, the exposure to 10 μM or 30 μM PTER decreased the peak amplitude of *I*_K(TO)_ to 387 ± 12 pA (*n* = 8, *p* < 0.05) or 256 ± 11 pA (*n* = 8, *p* < 0.05), respectively, from the control value of 517 ± 16 pA (*n* = 8). Concomitant with these data, the values for the inactivation time constant (τ) of *I*_K(TO)_ during membrane depolarization in the presence of 10 or 30 μM PTER were significantly reduced to 90.9 ± 8.2 ms (*n* = 8, *p* < 0.05) or 83.1 ± 6.2 ms (*n* = 8, *p* < 0.05), respectively, from the control value of 94.8 ± 9.8 ms (*n* = 8).

### 2.5. Effect of PTER on Ca^2+^-Activated K^+^ Current (I_K(Ca)_) in GH_3_ Cells

**Figure 6:**
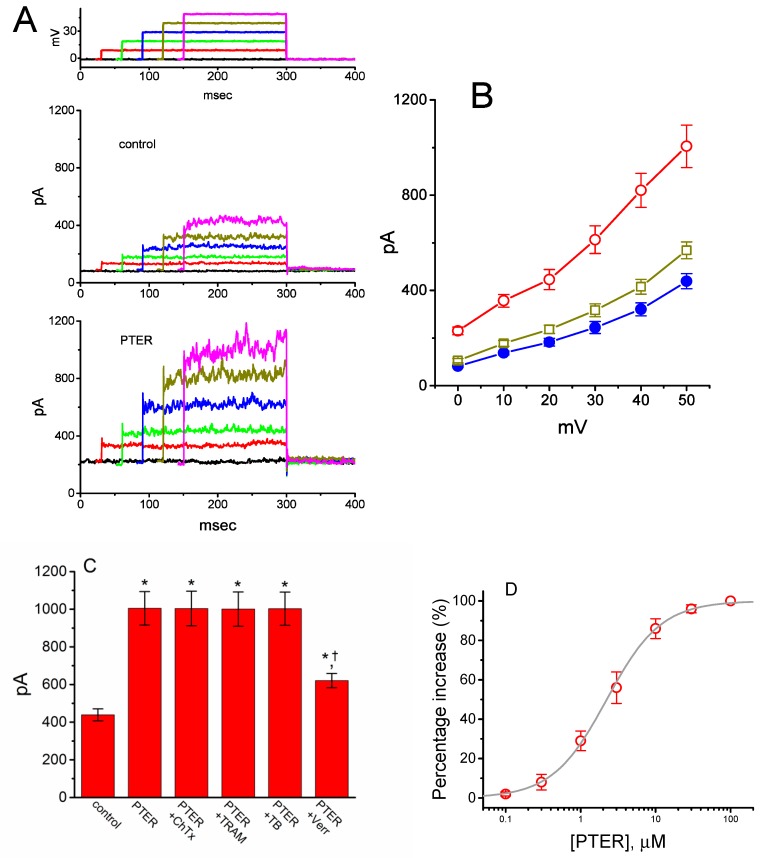
Stimulatory effect of PTER on Ca^2+^-activated K^+^ current (*I*_K(Ca)_) identified in GH_3_ cells. This set of whole-cell current recordings was conducted in cells suspended in normal Tyrode’s solution, which contained 1.8 mM CaCl_2_. (**A**) Representative *I*_K(Ca)_ traces obtained during the voltage steps to various membrane potentials between 0 and +50 mV in 10-mV steps from a holding potential of 0 mV (as indicated in the uppermost part of (**A**)). Records shown in the upper and lower panels were obtained under control conditions and during exposure to 3 μM PTER, respectively. The uppermost part depicts the voltage pulses applied. (**B**) Averaged *I*–*V* relations of *I*_K(Ca)_ obtained in the control (●), during exposure to 3 μM PTER (○), and after washout of the agent (□) (mean ± SEM; *n* = 8 for each data point). (**C**) Summary bar graph showing the effects of PTER, PTER plus chlorotoxin, PTER plus TRAM-39, PTER plus tolbutamide, and PTER plus verruculogen on *I*_K(Ca)_ amplitude (mean ± SEM; *n* = 8 for each bar). Current amplitude was measured at the end of the voltage step from 0 to +50 mV. PTER: 3 μM PTER; ChTx: 1 μM chlorotoxin; TRAM: 1 μM TRAM-39; TB: 10 μM tolbutamide; Verr: 1 μM verruculogen. *Significantly different from control (*p* < 0.05); †significantly different from the PTER (3 μM) alone group (*p* < 0.05). (**D**) Concentration-dependent effect of PTER on *I*_K(Ca)_ amplitude activated by membrane depolarization (mean ± SEM; *n* = 7–8 for each data point). Current amplitude was measured at the end of the depolarizing pulse from 0 to +50 mV. As cells were exposed to PTER (100 μM), current amplitude was taken to be 100%, and current amplitude achieved at different concentrations was then compared with the control value. The bold sigmoidal curve indicates a best fit to a modified Hill function, as detailed in Materials and Methods. The estimated values for EC_50_ and the Hill coefficient were 2.23 μM and 1.2, respectively.

Earlier observations have reported the ability of resveratrol to activate BK_Ca_ channels [29,30,31]. In the next set of experiments, we determined whether PTER could alter the amplitude of *I*_K(Ca)_ activated in response to membrane depolarization present in GH_3_ cells. In this set of whole-cell current recordings, we suspended GH_3_ cells in normal Tyrode’s solution containing 1.8 mM CaCl_2_, and the pipette used was filled with K^+^-containing solution, the composition of which is mentioned above. As cells settled down the bottom of the chamber, whole-cell current recordings were made. In an attempt to inactivate most voltage-gated K^+^ currents [51,52], we maintained the examined cells at the level of 0 mV, and then applied a series of voltage pulses between 0 and +50 mV with 10-mV steps. Within 1 min of exposing GH_3_ cells to PTER (3 μM), the amplitude of *I*_K(Ca)_ elicited by this voltage profile evidently rose (Figure 6A). For example, the addition of 3 μM PTER substantially increased the *I*_K(Ca)_ amplitude elicited by depolarizing pulse from 0 to +50 mV, from 568 ± 35 to 1005 ± 89 pA (*n* = 8, *p* < 0.05). As the agent was washed out, the current amplitude returned to 621 ± 38 pA (*n* = 8). The averaged *I*–*V* relationships of *I*_K(Ca)_ amplitude in the control, during the exposure to 3 μM PTER and after washout of the compound are depicted in Figure 6B. The addition of 3μM PTER substantially increased the whole-cell conductance of *I*_K(Ca)_ measured at the voltages between +30 and +50 mV to 19.6 ± 0.8 nS (*n* = 8, *p* < 0.05) from the control value of 12.6 ± 0.5 nS (*n* = 8). Moreover, as summarized in Figure 6C, *I*_K(Ca)_ amplitude increased by 3 μM PTER was suppressed by subsequent addition of verruculogen (1 μM), yet not by chlorotoxin (1 μM), TRAM-39 (3 μM) or tolbutamide (10 μM). Chlorotoxin or verruculogen is a blocker of Cl^−^ or BK_Ca_ channels, respectively, while TRAM-39 or tolbutamide was reported to inhibit intermediate-conductance Ca^2+^-activated K^+^ (IK_Ca_) channels or ATP-sensitive K^+^ (K_ATP_) channels, respectively [4]. For example, subsequent addition of verruculogen (1 μM), but still in the presence of 3 μM PTER, attenuated the *I*_K(Ca)_ amplitude from 1012 ± 92 to 621 ± 38 pA (*n* = 8, *p* < 0.05). Therefore, unlike its inhibitory action on *I*_K(TO)_, PTER could increase the amplitude of *I*_K(Ca)_ observed in these cells, and its stimulation of *I*_K(Ca)_ was largely attributable to the activation of large-conductance Ca^2+^-activated K^+^ (BK_Ca_) channels.

Figure 6D shows the relationship between the PTER concentration and the percentage increase of *I*_K(Ca)_. PTER could increase the amplitude of *I*_K(Ca)_ activated during membrane depolarization in a concentration-dependent manner. The half maximal concentration (EC_50_) required for the stimulatory effect of PTER on *I*_K(Ca)_ was 2.23 μM, and at a concentration of 100 μM, it fully increased the current amplitude. These results thus demonstrate the effectiveness of PTER in producing a stimulatory action on *I*_K(Ca)_ in GH_3_ cells.

### 2.6. Activity of Large-Conductance Ca^2+^-Activated K^+^ (BK_Ca_) Channels Stimulated by PTER in mHippoE-14 Neurons

**Figure 7:**
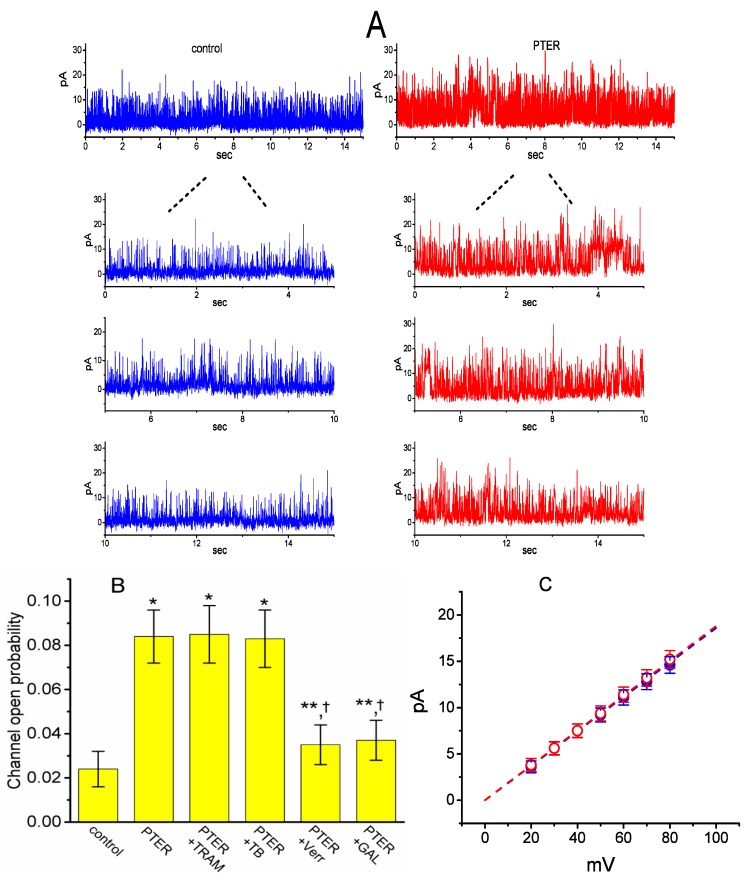
Stimulatory effect of PTER on the activity of large-conductance Ca^2+^-activated K^+^ (BK_Ca_) channels recorded from the detached patch of hippocampal mHippoE-14 neurons. In these experiments, cells were bathed in a high-K^+^ solution containing 0.1 μM Ca^2+^, and inside-out configuration with a holding potential of +60 mV was made. (**A**) Single-channel currents flowing through BK_Ca_ channels obtained under control conditions (i.e., PTER was not present, left) and after bath application of 3 μM PTER (i.e., at the cytosolic face of the detached patch, right). The lower portions in each panel indicate the expanded records of the uppermost part in the absence (left) and presence (right) of 3 μM PTER. Upward deflections represent channel openings. (**B**) Summary bar graph depicting the effects of PTER, PTER plus TRAM-39, PTER plus tolbutamide, PTER plus verruculogen, and PTER plus GAL-021 (mean ± SEM; *n* = 8–10 for each bar). Inside-out current recordings with a holding potential of +60 mV were performed to measure the channel opening probability. PTER: 3 μM pterostilbene; TRAM: 3 μM TRAM-39; TB: 10 μM tolbutamide; Verr: 1 μM verruculogen; GAL-021: 10 μM GAL-021. * or **: Significantly different from control (*p* < 0.01 or *p* < 0.05, respectively); †significantly different from the PTER (3 μM) alone group (*p* < 0.05). (**C**) Averaged *I*–*V* relations of BK_Ca_ channels in the absence (●) and presence (○) of 3 μM PTER (mean ± SEM; *n* = 8 for each data point). In inside-out current recordings, single-channel amplitude was measured at the level of each holding potential. Of note, the single-channel conductances (indicated by a dashed line) in the absence and presence of PTER are virtually superimposable with each other.

We next tested the question of whether the presence of PTER could perturb the single-channel currents flowing through BK_Ca_ channels identified in these cells [53]. As depicted in Figure 7A, under inside-out current recordings, when PTER at a concentration of 3 μM was applied to the intracellular leaflet of the detached patch, the probability of BK_Ca_ channels that would be open substantially rose, as evidenced by a significant increase in channel activity to 0.084 ± 0.012 (*n* = 10, *p* < 0.01) from a control value of 0.024 ± 0.008 (*n* = 10), although minimal change in single-channel current was noticeable in the presence of PTER. After washout of the agent, BK_Ca_-channel activity returned to 0.029 ± 0.009 (*n* = 7, *p* < 0.01).

### 2.7. Comparisons of the Effects of PTER, PTER Plus TRAM-39, PTER Plus Tolbutamide, PTER Verruculogen, and PTER Plus GAL-021 on the Probability of BK_Ca_-Channel Openings in mHippoE-14 Neurons

We further evaluated whether the stimulatory effect of PTER on BK_Ca_-channel activity can be altered by subsequent application of TRAM-39, tolbutamide, verruculogen or GAL-021. In these experiments, in which cells were bathed in a high-K^+^ solution containing 0.1 μM Ca^2+^, inside-out patch configuration was made and the potential was constantly held at +60 mV. As summarized in Figure 7B, in the continued presence of 3 μM PTER, subsequent addition of neither TRAM-39 (3 μM) nor tolbutamide (10 μM) substantially modified PTER-mediated elevation in the channel opening probability, while that of verrculogen (1 μM) or GAL-021 (10 μM) was capable of reversing the PTER-induced raise in channel activity. GAL-021 is recognized as an intravenous inhibitor of BK_Ca_ channels [54]. For example, further application of GAL-021 (10 μM), but still in the presence of PTER (3 μM), attenuated channel activity from 0.084 ± 0.012 to 0.037 ± 0.009 (*n* = 8, *p* < 0.05).

### 2.8. Lack of PTER Effect on Single-Channel Conductance of BK_Ca_ Channels in mHippoE-14 Neurons

The activity of BK_Ca_ channels at different levels of membrane potentials obtained with or without PTER addition was further assessed and compared in these cells. Figure 6B depicts *I*–*V* relations of BK_Ca_ channels established under control conditions and during exposure to PTER (3 μM). The single-channel conductance of BK_Ca_ channels derived from the linear *I*–*V* relation in the control (i.e., when PTER was not present) was 186 ± 9 pS (*n* = 8), a value that did not differ significantly from that achieved after the application of PTER (188 ± 7 pS; *n* = 8, *p* > 0.05). It is clear, therefore, from these data that the presence of PTER resulted in no considerable change in the single-channel conductance of the channel; however, it did increase the open-state probability of BK_Ca_ channels in mHippoE-14 neurons.

## 3. Discussion

The principal findings from the present study are as follows. First, in pituitary GH_3_ cells, the addition of PTER inhibited *I*_h_ effectively in a concentration- and time-dependent manner. Second, the steady state activation curve of *I*_h_ was distinctly shifted to more hyperpolarizing potentials by 11 mV, producing channel opening at more negative voltages. Third, PTER at a concentration higher than 10 μM was able to suppress the amplitude of *I*_Ca,l_ and *I*_K(TO)_. Fourth, its presence substantially raised the amplitude of macroscopic *I*_K(Ca)_. Fifth, in hippocampal mHippoE-14 neurons, under inside-out patch recordings, the addition of PTER failed to change the single-channel conductance of BK_Ca_ channels; however, it did increase the probability of the channels that would be opened. The present observations, therefore, reflect that the effects on the modifications of ion-channel activity could conceivably be one of the ionic mechanisms underlying PTER-induced actions, if similar in vitro or in vivo results can emerge in central neurons (e.g., mHippoE-14 neurons), and in neuroendocrine or endocrine cells (e.g., GH_3_ cells).

Previous studies have shown that stilbenes might increase the sensitivity of striatal muscarinic receptors and suppress the activity of acetylcholinesterase [20,25]. PTER analogs were also recently reported to be antagonists of nicotinic acetylcholine receptors [55]. However, in the continued presence of PTER, further application of atropine, a blocker of muscarinic receptors, or nicotine, an activator of nicotinic receptors, was unable to modify PTER-mediated inhibition of *I*_h_ in GH_3_ cells. Thus, PTER-induced blocking of *I*_h_ or stimulation of *I*_K(Ca)_ seen in GH_3_ cells could not be predominantly attributed to either an increase in acetylcholine level or its binding to cholinergic receptors.

Four mammalian subtypes (HCN1, HCN2, HCN3, and HCN4) have been cloned to date [36,37,43,56]. Previous work has mentioned that HCN2, HCN3, or mixed HCN2 + HCN3 channels could be functionally expressed in GH_3_ cells or various types of central neurons and endocrine or neuroendocrine cells [35,37,38,39,57]. Because of the crucial roles of *I*_h_ (i.e., HCNx-encoded currents) in contributing to the excitability and automaticity existing in various types of electrically excitable cells [33,35,37,38,39,42,49,57], findings from this study could provide substantial insights into the electrophysiological and pharmacological properties of PTER and other structurally related compounds.

It has previously been demonstrated that long-term treatment with PTER might be able to suppress cholesterol biosynthesis, thus causing the intracellular accumulation of oxysterols [58]. Depletion of membrane cholesterol with methyl-β-cyclodextrin was previously reported to increase the activity of large-conductance Ca^2+^-activated K^+^ (BK_Ca_) channels [59]. Membrane cholesterol content was also shown to modify the amplitude of HCN-encoded currents [56]. However, the PTER-induced blocking of *I*_h_ observed in GH_3_ cells is rapid in onset and hence appears to be unlinked to its suppression in the conversion of 7-dehydrocholesterol to cholesterol occurring inside the cell.

The addition of PTER was observed to enhance the amplitude of *I*_K(Ca)_ in GH_3_ cells. One would expect that *I*_K(Ca)_ amplitude stimulated by PTER could result from the elevation of *I*_Ca,l_ flowing through l-type Ca^2+^ channels. However, in this study, the presence of PTER at a concentration higher than 10 μM decreased the peak amplitude of *I*_Ca,l_ in response to membrane depolarization. It therefore seems unlikely that the PTER stimulation of macroscopic *I*_K(Ca)_ presented herein ascribes indirectly from an increase in intracellular Ca^2+^ concentration through elevation of membranous Ca^2+^-channel activity. In keeping with these observations, BK_Ca_-channel activity observed in the detached patches of mHippoE-14 neurons was substantially raised after bath application of PTER.

Resveratrol was previously reported to stimulate BK_Ca_-channel activity [29,31]. In the continued presence of PTER (3 μM), further addition of 3 μM resveratrol did not produce any stimulatory effect on the probability of BK_Ca_-channel openings in mHippoE-14 neurons. The present results demonstrate that the stimulatory effect of PTER and resveratrol on the activity of BK_Ca_ channels was not additive, suggesting that these two compounds, which are structurally related, may functionally interact with similar binding sites existing in the BK_Ca_ channel.

Stilbenoids, including PTER, have the ability to modulate the magnitude of GABA-induced Cl^−^ currents [60]. However, in the current study, the PTER-mediated increase in *I*_K(Ca)_ amplitude was not substantially affected by further addition of either chlorotoxin (1 μM), a blocker of Cl^−^ channels, or tolbutamide (10 μM), an inhibitor of K_ATP_ channels, or TRAM-39 (3 μM), an inhibitor of IK_Ca_ channels. Therefore, the possibility proposing that PTER-stimulated *I*_K(Ca)_ observed in GH_3_ cells or hippocampal mHippoE-14 neurons is largely mediated through its modulation of Cl^−^-, K_ATP_- or IK_Ca_-channel activity would be virtually excluded.

Besides the suppression of *I_h_* amplitude activated in response to membrane hyperpolarization, the activation time course of the current progressively became slowed during the exposure to PTER. In other words, the blocking of *I*_h_ produced by PTER is apparently not instantaneous, but it does develop over time after HCN channel(s) become opened (i.e., *I*_h_ was activated) upon long-lasting membrane hyperpolarization. It is also noted that the τ value of *I*_K(TO)_ inactivation in response to membrane depolarization was increased in the presence of PTER higher than 10 μM. It is reasonable to assume that the PTER molecules exhibit a low affinity for the resting state and a high affinity for the activated state of the channel(s) in GH_3_ cells. The blocking site of PTER thus appears to be located within the channel pore only when the channel is open (i.e., when macroscopic *I*_h_ is activated). However, to what extent the PTER-induced block of *I*_h_ is linked to its ability to form hydrogen bonds and to generate stable resonance structures in the presence of oxidative electrophilic molecules, possibly in or around the HCN channels, will deserve vigorous further study.

It needs to be emphasized that the PTER addition to GH_3_ cells produced significant depressant action on the amplitude of *I*_h_ in a concentration-dependent manner, with an IC_50_ value of 0.84 μM, a value that is slightly lower than the EC_50_ value (i.e., 2.23 μM) required for its stimulation of *I*_K(Ca)_. No discernible modification in the single-channel conductance of BK_Ca_ channels in mHippoE-14 neurons was demonstrated; however, it did enhance the probability of BK_Ca_-channel openings. By use of a two-step voltage protocol, exposure to PTER (1 μM) could also distinctly shift the steady state activation curve of *I*_h_ along the voltage axis in a leftward direction by 11 mV. Therefore, the sensitivity of *I*_h_ to PTER could be dependent on the PTER concentration achieved, the level of resting potential, the firing rate of APs, or a combination. The serum PTER level measured in rats was reported to reach 0.12 μM [25]. The actions of PTER or other stilbenoids on ionic currents presented herein could have characteristics that make it significant from a therapeutic or toxicological standpoint. To this end, PTER-mediated perturbations of *I*_h_, *I*_K(Ca)_ or BK_Ca_-channel activity are of particular importance, and they tend to be upstream of its actions on aberrant signaling pathways including regulation in DNA methylation, inhibition of telomerase activity, or other anti-neoplastic or anti-oxidative activities [7,10,16,18,19,58,61,62,63,64].

## 4. Materials and Methods

### 4.1. Chemicals, Drugs and Solutions

Pterostilbene (PTER, trans-3,5-dimethoxy-4-hydroxystilbene), atropine, nicotine, resveratrol (3,4′,5-trihydroxy-trans-stilbene), tetraethylammonium chloride (TEA), tetrodotoxin (TTX) and tolbutamide were acquired from Sigma-Aldrich (Merck, Taipei, Taiwan), while TRAM-39 (2-chloro-α,α-diphenylbenzeneacetonitrile) was from Tocris (Union Biomed Inc., Taipei, Taiwan). Verruculogen was obtained from Alomone (Asia Bioscience, Taipei, Taiwan), and GAL-021 (N^2^-methoxy-N^2^-methyl-N^4^,N^6^-dipropyl-1,3,5-triazine-2,4,6-triamine) was from MedChemExpress (Everything Biotech Ltd., New Taipei City, Taiwan), while SDZ-202791 was from Santa Cruz (Hong Jing, New Taipei City, Taiwan). Chlorotoxin was a kind gift provided by Professor Dr. Woei-Jer Chuang (Department of Biochemistry, National Cheng Kung University Medical College, Tainan City, Taiwan). Cell culture media, l-glutamine, horse serum, and fetal bovine or calf serum were generally obtained from HyClone™ (Thermo Fisher Scientific, Logan, UT, USA), whereas other chemicals, including CsCl, CsOH, aspartic acid, EGTA, and HEPES, were commercially available and of analytical reagent grade.

The composition of the standard extracellular solution (i.e., normal Tyrode’s solution), in which cells were suspended, was as follows (in mM): NaCl 136.5, KCl 5.4, CaCl_2_ 1.8, MgCl_2_ 0.53, glucose 5.5, and HEPES-NaOH buffer 5.5 (pH 7.4). To measure *I*_h_ or *I*_K(Ca)_ and preclude contamination of Cl^−^ currents, the patch pipette was backfilled with a solution (in mM) containing: K-aspartate 130, KCl 20, KH_2_PO_4_ 1, MgCl_2_ 1, Na_2_ATP 3, Na_2_GTP 0.1, EGTA 0.1, and HEPES-KOH buffer 5 (pH 7.2). To record *I*_Ca,l_, the pipette solution contained (in mM): CsCl 130, EGTA 0.1, MgCl_2_ 1, Na_2_ATP 3, Na_2_GTP 0.1 and HEPES-CsOH buffer 5 (pH 7.2). For the recordings of BK_Ca_-channel activity, the bath solution was replaced with a high K^+^ solution (in mM) containing: KCl 145, MgCl_2_ 0.53, and HEPES-KOH buffer 5 (pH 7.2), and the pipettes were filled with a solution (in mM) consisting of KCl 145, MgCl_2_ 2, and HEPES-KOH buffer 5 (pH 7.4). All solutions were prepared using deionized water from a Milli-Q water purification system (APS Water Services, Inc., Van Nuys, CA, USA), and both the pipette solution and culture medium used were commonly filtered on the day of use with a sterile Acrodisc^®^ filter with a 0.2-μm Supor^®^ membrane (Pall Corp., Port Washington, NY, USA).

### 4.2. Cell Preparations

GH_3_ pituitary tumor cells, acquired from the Bioresources Collection and Research Center ([BCRC-60015]; Hsinchu, Taiwan; originally derived from ATCC [CCL-82.1]), were maintained in Ham’s F-12 medium supplemented with 15% horse serum (*v/v*), 2.5% fetal calf serum (*v/v*), and 2 mM l-glutamine. An established embryonic mouse hippocampal cell line (mHippoE-14; CLU198) was acquired from Cedarlane (Burlington, ON, Canada) [65,66,67,68]. mHippoE-14 cells were maintained in Dulbecco’s modified Eagle’s medium supplemented with 10% fetal bovine serum and 2 mL l-glutamine. GH_3_ or mHippoE-14 cells were plated as a monolayer culture on 50-mL plastic culture flasks in a humidifier environment of 5% CO_2_/95% air at 37 °C. The medium was refreshed every 2 days to maintain a healthy cell population. Under our experimental conditions, the presence of neurites and varicosities during the preparations of mHippoE-14 neurons could often be observed. The recordings were performed 5 or 6 days after cells were subcultured (60%–80% confluence).

### 4.3. Electrophysiological Measurements

Prior to each experiment, cells (i.e., GH_3_ cells and mHippoE-14 neurons) were gently dissociated, and we transferred an aliquot of cell suspension to a home-made chamber mounted on the fixed stage of an inverted TMS-F microscope (Nikon, Tokyo, Japan). The microscope was coupled to a video camer system with magnification up to 1500×. Cells were immersed at room temperature (20–25 °C) in normal Tyrode’s solution. The patch electrodes used were drawn from Kimax-51 glass capillaries (#34500; Kimble, Vineland, NJ, USA) on a PP-830 vertical puller (Narishige, Tokyo, Japan), and their tips fire-polished with MF-83 microforge (Narishige). When the electrodes were filled with different internal solutions, their resistances generally ranged from 3 to 5 MΩ. A three-dimensional oil-driven micromanipulator (MO-103; Narishige, Tokyo, Japan) was used to precisely position the electrode near the cell examined. Ionic currents were measured in the whole-cell, cell-attached or inside-out configuration of the patch-clamp technique by using an RK-400 patch-clamp amplifier (Bio-Logic, Claix, France) [51,52]. The liquid junction potentials were generally nulled shortly before seal formation was made, and the whole-cell results were corrected by the potentials measured under our experimental conditions.

### 4.4. Data Recordings

The signals, comprising both potential and current traces, were acquired through analog-to-digital conversion and monitored on the organic light-emitting diode (OLED) display of an ASUS VivoBook Flip-14 touch-screen laptop computer (TP412U; Taipei City, Taiwan). The records were stored online at 10 kHz on the computer, connected with a Digidata 1440A interface (Molecular Devices, Sunnyvale, CA, USA). During the recordings, the latter device was controlled by the pCLAMP 10.7 software package (Molecular Devices). Current signals obtained were low-pass filtered at 2 kHz with an FL-4 four-pole Bessel filter (Dagan, Minneapolis, MN, USA). Through digital-to-analog conversion, pCLAMP-generated profiles comprising different rectangular or linear ramp waveforms were designed to acquire data for constructing either the current versus voltage (*I*–*V*) relationship or the activation curve of *I*_h_. After the digital data were collected, we later analyzed them using various analytical tools including the LabChart 7.0 program (AD Instruments; Gerin, Tainan, Taiwan), 64-bit OriginPro 2016 (Microcal, Northampton, MA, USA), or custom-built macros run under Microsoft Excel™ 2016 (Redmond, WA, USA).

### 4.5. Data Analyses

To evaluate the concentration-dependent inhibition of PTER on the amplitude of *I*_h_, the cells were immersed in Ca^2+^-free Tyrode’s solution, the cell examined was held at −40 mV, and a 2-s hyperpolarizing pulse to −110 mV was driven to activate *I*_h_. Current amplitudes at the end of each hyperpolarizing pulse were measured under control conditions and during cell exposure to different PTER concentrations (0.1–30 μM). The concentration required to suppress 50% of *I*_h_ amplitude was determined with the goodness of fit by using a modified Hill function (Equation (1)):

(1) $$\mathrm{Relative}\;\mathrm{amplitude}=\frac{\left(1-a\right)\times {\left[PTER\right]}^{-n_{H}}}{{\left[PTER\right]}^{-n_{H}}+IC_{50}^{-n_{H}}}+a$$

where [PTER] is the PTER concentration applied; IC_50_ and n_H_ are the concentration required for a 50% inhibition and the Hill coefficient, respectively; and maximal inhibition (i.e., 1 − *a*) was also estimated in this equation.

To determine the inhibitory action of PTER on the voltage dependence of *I*_h_, the quasi-steady state activation curve of the current was achieved by using a two-step protocol. The relationships between the conditioning voltage pulses and the normalized amplitudes of *I*_h_ with and without PTER (1 μM) addition are described and were hence fitted by a Boltzmann function (Equation (2)) in the following form:

(2) $$I=\frac{I_{max}}{1+exp\left[\frac{\left(V-V_{\frac{1}{2}}\right)qF}{RT}\right]}$$

where *I*_max_ is the maximal activated *I*_h_, *V* the conditioning potential, *V*_1/2_ the membrane for half-maximal activation, *q* the apparent gating charge, and *F*, *R*, or *T* is Faraday’s constant, the universal gas constant or the absolute temperature, respectively.

To assess the concentration-dependent effect of PTER on *I*_K(Ca)_ amplitude, each cell was depolarized to +50 mV from a holding potential of 0 mV. Current amplitude measured at the end of depolarizing voltage during cell exposure to 100 μM PTER was considered to be 100%. Mean values in then concentration-dependent relation of PTER to the stimulation of *I*_K(Ca)_ were least-squares fitted to the Hill equation (Equation (3)). That is,

(3) $$\mathrm{percentage}\;\mathrm{increase}\;\left(\%\right)=\frac{E_{\max}}{1+\frac{{\mathrm{EC}}_{50}^{n_{H}}}{{\left[\mathrm{PTER}\right]}^{n_{H}}}}$$

where [PTER] represents the concentration of PTER, EC_50_ is the concentration that produces 50% of maximal stimulation, n_H_ is the Hill coefficient, and E_max_ is the PTER-induced maximal stimulation of *I*_K(Ca)_.

### 4.6. Single-Channel Analyses

Single-channel currents flowing through BK_Ca_ channels measured from surface membranes of GH_3_ cells were recorded and analyzed using pClamp 10.7 (Molecular Devices). Single-channel amplitudes were commonly evaluated by fitting Gaussian distributions to the amplitude histograms of the closed and open states. Channel activity was defined as *N*·*P*_O_, namely the product of the channel number (*N*) and open probability (*P*_O_). Single-channel conductance was calculated by linear regression using mean values of current amplitudes measured at the different levels of the holding potentials.

### 4.7. Statistical Analyses

The data regarding macroscopic and single-channel currents were collected and are provided as the mean values ± standard error of the mean (SEM), with sample sizes (*n*) indicating the number of cells used to analyze the experimental results, and error bars were plotted as SEM. We made assertions about the variability of means that could be collected from a random cohort derived from the population concerned; hence, the SEM could be more appropriate than the standard deviation. The paired or unpaired Student’s *t*-test or a one-way analysis of variance (ANOVA) followed by post-*hoc* Fisher’s least-significance difference test for multiple comparisons were commonly implemented for statistical evaluation. However, as the assumption of normality underlying ANOVA was possibly violated, we used the non-parametric Kruskal–Wallis test. Values of *p* < 0.05 were regarded as statistically significant, unless stated otherwise.

## Abbreviations

AP	Action potential
BK_Ca_	Channel large-conductance Ca^2+^-activated K^+^ channel
EC_50_	The concentration required for 50% stimulation
HCN channel	Hyperpolarization-activated cyclic nucleotide-gated ion channel
IC_50_	The concentration required for 50% inhibition
I_Ca,l_	l-type Ca^2+^ current
I_h_	Hyperpolarization-activated cation current
I_K(Ca)_	Ca^2+^-activated K^+^ current
I_K(M)_	M-type K^+^ current
I_K(TO)_	Transient outward K^+^ current
I–V	Current versus voltage
PTER	Pterostilbene (trans-3,5-dimethoxy-4′-hydroxystilbene)
SEM	Standard error of the mean
τ	Time constant
TEA	Tetraethylammonium chloride
TTX	Tetrodotoxin
